# Correlation of the Neutrophil-to-Lymphocyte Ratio (NLR) and Platelet-to-Lymphocyte Ratio (PLR) with Contrast-Induced Nephropathy in Patients With Acute Coronary Syndrome Undergoing Percutaneous Coronary Interventions

**DOI:** 10.7759/cureus.11879

**Published:** 2020-12-03

**Authors:** Khurram Butt, Jason D'Souza, Cai Yuan, Jayapriya Jayakumaran, Michelle Nguyen, Hamza I Butt, Khalid Abusaada

**Affiliations:** 1 Internal Medicine, AdventHealth Orlando, Orlando, USA; 2 Cardiology, St. Luke's Health System, University of Missouri, Kansas City, USA; 3 Oncology, University of Florida, Gainesville, USA; 4 Internal Medicine, Ocala Regional Medical Center, University of Central Florida College of Medicine, Orlando, USA; 5 Internal Medicine, Ocala Regional Medical Center, University of Central Florida College of Medicine, Ocala, USA; 6 Epidemiology and Public Health, Mel and Enid Zuckerman College of Public Health, University of Arizona, Tucson, USA

**Keywords:** cardiac catheterization, nephropathy, percutaneous coronary intervention (pci), neutrophil to lymphocyte ratio (nlr), coronary artery disease (cad), contrast-induced nephropathy (cin), acute myocardial infarction (ami), acute kidney injury (aki), platelet to lymphocyte ratio (plr)

## Abstract

Introduction

Contrast-induced acute nephropathy (CIN) in patients undergoing percutaneous coronary intervention (PCI) in the setting of acute coronary syndromes (ACS) is associated with adverse outcomes, including longer hospitalization and short and long-term mortality. Neutrophil to lymphocyte ratio (NLR) and platelet to lymphocyte ratio (PLR) are inflammatory markers that have been validated separately in prior studies as a predictor of CIN in patients with ACS who undergo a left heart catheterization. Our study aims to further investigate the role of NLR and PLR together as markers for predicting CIN in patients with ACS.

Methods

A retrospective chart review was performed on a total of 1,577 patients aged 18 - 90 who presented with ACS and underwent PCI between January 2011 to December 2015 at the Florida Hospital Orlando. Cut-off values used for a high PLR and NLR were PLR > 128 and NLR > 2.6. CIN was defined as an increased serum creatinine level by ≥ 0.5 mg/dL, or ≥ 25%, over the baseline value within 72 hours after contrast agent administration. Patients with end-stage renal disease (ESRD) were excluded.

Results

Of the 1,577 patients included in the study, 213 (13.51%) patients had CIN. On multivariate logistic regression analysis, high NLR showed an independent association with an elevated risk of CIN (OR 2.03, 95% CI: 1.403 - 3.176, P < 0.001). High PLR did not correlate with CIN (OR 0.831, 95% CI: 0.569 - 1.214, P = 0.339).

Conclusion

Elevated NLR is an independent predictor of CIN in patients with acute myocardial infarction (AMI) and may be used to improve on current risk prediction models.

## Introduction

Contrast-induced nephropathy (CIN) is the third leading cause of in-hospital acute kidney injury (AKI) [1] and is associated with significant in-hospital and long-term morbidity and mortality [2-3]. The pathogenesis of CIN is multifactorial and multiple mechanisms have been suggested; inflammation and prothrombotic milieu are known to play a key role. Hence, mediators reflecting inflammation and thrombosis, such as neutrophil to lymphocyte ratio (NLR) and platelet to lymphocyte ratio (PLR), are being studied as markers of CIN [4]. The interest in the association between NLR and PLR and the risk of cardiovascular events in patients undergoing angiography or percutaneous coronary intervention (PCI) is rapidly growing. Multiple studies have established an association of PLR and NLR with the risk of CIN in ST-elevation myocardial infarction (STEMI) and non-ST-elevation myocardial infarction (NSTEMI) patients [5-7]. However, the utility of these markers in combination with other factors in predicting CIN in acute myocardial infarction (AMI) has not been studied. In this study, we assessed the correlation between NLR and PLR and CIN in patients with AMI and to evaluate the effect of these scores on the performance of the Mehran score to predict the risk of CIN in this population [8].

## Materials and methods

This was a retrospective analysis of data collected as part of the Acute Coronary Treatment and Intervention Outcomes Network (ACTION) Registry® (now called the Chest Pain-MI Registry™) [9] at a tertiary care center between January 1, 2011 and December 31, 2015. Data in the Registry is collected prospectively for patients who present within 24 hours of the onset of an ischemic event with a primary diagnosis of myocardial infarction (STEMI or NSTEMI). In addition, the research team conducted a chart review of all included patients to obtain the following data that is not collected as part of the Chest Pain-MI Registry: absolute neutrophil and lymphocyte counts, platelet count, the highest creatinine recorded within 72 hours of left heart catheterization and PCI, contrast volume, and the use of intra-aortic balloon pump (IABP). The study was approved by the Advent Health Institutional Review Board (approval 868065). Patients were excluded if they did not undergo a PCI, were receiving long-term renal replacement therapy prior to admission, or if no creatinine level was obtained within 72 hours of the PCI.

NLR and PLR were defined as the ratio of the absolute neutrophil count and the absolute platelet count, respectively, to the absolute lymphocyte count. CIN was defined as a rise in the serum creatinine level by ≥ 0.5 mg/dL or ≥ 25% over the baseline value within 72 hours after the contrast agent was administered. Cardiogenic shock is defined as having one or more of the following conditions: (a) a sustained decline of > 30 minutes of systolic blood pressure to < 90 mm Hg or a cardiac index < 2.2 L/min/m^2^; (b) requirement for parenteral inotropic/vasopressor agents or mechanical support to maintain a systolic blood pressure ≥ 90 mm Hg or cardiac index ≥ 2.2 L/min/m^2^. Tachycardia was defined as a heart rate on admission of > 100 beats per minute.

Association of AKI and NLR or PLR was evaluated using the Student t-test. NLR and PLR were categorized using cut-off values established by Cho et al. [10]. An NLR level over 2.6 and a PLR level over 128 were considered high. Baseline characteristics of patients with high NLR and PLR levels were compared to those with low NLR and PLR levels, respectively, in a bivariate analysis using the Student’s t-test for continuous variables and the Chi-square test for categorical variables. A two-sided p-value of ≤ 0.05 was considered statistically significant.

A multivariate logistic regression model was built with CIN as the outcome and the following variables as covariates: PLR, NLR, left ventricular ejection fraction, presence of acute congestive heart failure (CHF) at time of admission, presence of shock at the time of admission, cardiac arrest at the time of admission, diabetes, hypertension, age, gender, glomerular filtration rate (GFR), anemia, tachycardia, and the use of IABP.

Finally, we evaluated the contribution of NLR to the prediction of CIN in patients with AMI through comparison of the area under receiver operating characteristic curve (AUROC) and calibration between a Mehran score model and a Mehran score model, plus categorized NLR. The Mehran score was calculated based on the factors reported by Mehran et al. [8]. A two-sided p-value of ≤ 0.05 was considered statistically significant for all comparisons. The data analysis was performed using Stata, version 13.0 (StataCorp LLC, College Station, TX, USA).

## Results

A total of 1,577 patients were included in the analysis. Two hundred and thirteen (13.51%) patients developed CIN. Patients with CIN had a higher NLR (6.9 vs 4.5, p = 0.003) and a higher PLR (164 vs 133, p < 0.001). High NLR was associated with CIN (p < 0.001), STEMI patients (p = 0.008), anemia (p < 0.001), CHF admission (p = 0.009), cardiac arrest on admission (p = 0.026), diabetes mellitus (p = 0.002), hypertension (p = 0.038), reduced left ventricular ejection fraction (LVEF) (p < 0.001), and tachycardia (p < 0.001) (Table 1). High PLR was associated with CIN (p = 0.002), male gender (p = 0.025), STEMI (p = 0.001), anemia (p < 0.001), CHF on admission (p = 0.023), cardiac arrest on admission (p = 0.011), and hypertension (p = 0.025) (Table 2).

**Table 1:** Bivariate Analysis of Demographic and Clinical Parameters with Neutrophil to Lymphocyte Ratio (NLR) Category CHF: congestive heart failure; CIN: contrast-induced acute kidney; DM: diabetes mellitus; HTN: hypertension; GFR: glomerular filtration rate; IABP: intra-aortic balloon pump; LVEF: left ventricular ejection fraction; SD: standard deviation; STEMI: ST-elevation myocardial infarction

Parameter	NLR ≤ 2.8	NLR > 2.8	p-value
Age (years), mean ± SD	61.10 ± 0.47	63.98 ± 0.43	1.000
CIN, n (%)	58 (8.45%)	155 (16.95%)	< 0.001
Male gender, n (%)	482 (70.26%)	626 (70.26%)	0.999
STEMI, n (%)	381 (55.55%)	435 (48.82%)	0.008
Anemia, n (%)	106 (15.45%)	202 (22.67%)	< 0.001
CHF at admission, n (%)	31 (4.52%)	69 (7.74%)	0.009
Shock admission, n(%)	5 (0.73%)	11 (1.23%)	0.320
Cardiac arrest at admission, n(%)	39 (5.69%)	30 (3.37%)	0.026
DM, n (%)	210 (30.61%)	338 (37.93%)	0.002
HTN, n (%)	492 (71.72%)	680 (76.32%)	0.038
IABP, n (%)	17 (2.48%)	35 (3.93%)	0.110
GFR (ml/min), mean ± SD	76.09 ± 0.87	74.11 ± 0.85	0.055
LVEF, mean ± SD	50.94 ± 0.42	48.14 ± 0.41	< 0.001
Tachycardia, n (%)	107 (15.60%)	207 (23.23%)	< 0.001
Mortality, n (%)	20 (2.92%)	32 (3.59%)	0.456

**Table 2:** Demographic and Clinical Parameters by Platelet to Lymphocyte Ratio (PLR) Category CHF: congestive heart failure; CIN: contrast-induced acute kidney; DM: diabetes mellitus; HTN: hypertension; GFR: glomerular filtration rate; IABP: intra-aortic balloon pump; LVEF: left ventricular ejection fraction; SD: standard deviation; STEMI: ST-elevation myocardial infarction

Variables	PLR ≤ 128	PLR > 128	p-value
Age (years), mean ± SD	61.42 ± 0.40	64.73 ± 0.52	< 0.001
CIN, n (%)	109 (11.19%)	104 (16.53%)	0.002
Gender, male, n (%)	693 (72.34%)	415 (67.04%)	0.025
STEMI, n (%)	527 (55.01%)	289 (46.69%)	0.001
Anemia, n (%)	148 (15.45%)	160 (25.85%)	< 0.001
CHF at admission, n (%)	50 (5.22%)	50 (8.08%)	0.023
Shock at admission, n (%)	11 (1.15%)	5 (0.81%)	0.510
Cardiac arrest at admission, n (%)	52 (5.43%)	17 (2.75%)	0.011
DM, n (%)	321 (33.51%)	227 (36.67%)	0.197
HTN, n (%)	693 (72.34%)	479 (77.38%)	0.025
Use of IABP, n (%)	29 (3.03%)	23 (3.72%)	0.455
GFR (ml/min), mean ± SD	75.50 ± 0.75	74.18 ± 1.05	0.146
LVEF, mean ± SD	49.65 ± 0.38	48.94 ± 0.48	0.121
Tachycardia, n (%)	164 (17.12%)	150 (24.23%)	0.001
Mortality, n (%)	32 (3.34%)	20 (3.23%)	0.906

Multivariable logistic regression showed that after adjusted for other covariates, high NLR remained significantly associated with increased risk of CIN (OR 2.03, 95% CI 1.403-3.176, P<0.001) whilst high PLR was not (OR 0.831, 95% CI 0.569-1.214, P=0.339). Other factors significantly associated with increased risk of CIN were age, STEMI, shock on admission, cardiac arrest on admission, tachycardia, diabetes mellitus, hypertension, use of IABP, and anemia (Table 3). NLR level >2.6 had a sensitivity of 72%, specificity of 46%, positive predictive value of 17% and negative predictive value of 92%.

**Table 3:** Multivariable Logistic Regression Model for Prediction of Contrast-induced Nephropathy CHF: congestive heart failure; CIN: contrast-induced acute kidney; DM: diabetes mellitus; HTN: hypertension; GFR: glomerular filtration rate; IABP: intra-aortic balloon pump; LVEF: left ventricular ejection fraction; NLR: neutrophil to lymphocyte ratio; PLR: platelet to lymphocyte ratio; STEMI: ST-elevation myocardial infarction

Variables	Odds ratio	95% Confidence Interval	p-value
STEMI	1.432	1.027 - 1.998	0.034
GFR	1.007	1.000 - 1.012	0.056
LVEF	0.994	0.981 - 1.007	0.363
Anemia	1.865	1.302 - 2.674	0.001
NLR > 2.6	2.032	1.363 - 3.028	< 0.001
PLR > 128	0.858	0.590 - 1.247	0.422
CHF admission	1.652	0.971 - 2.809	0.064
Shock at admission	3.574	1.042 - 12.27	0.043
Cardiac arrest at admission	2.072	1.030 - 4.166	0.041
DM	1.688	1.385 - 2.706	0.002
HTN	2.149	1.320 - 3.496	0.002
Age	1.018	1.005 - 1.032	0.007
Tachycardia	1.721	1.200 - 2.468	0.003
Use of IABP	2.851	1.458 - 5.576	0.002

To evaluate the utility of NLR in the prediction of CIN, we incorporated the categorized NLR into the Mehran score model (Table 4). All factors included in the Mehran score were significant predictors of CIN, except contrast volume. When NLR was added to the model, it was an independent significant predictor of CIN (OR 1.80, p < 0.001). The new model with the addition of NLR had better discrimination ability with AUROC (0.73 vs 0.71, p = 0.015).

**Table 4:** Mehran Model With and Without High Neutrophil to Lymphocyte Ratio (NLR) CHF: congestive heart failure; CI: confidence interval; CKD: chronic kidney disease; DM: diabetes mellitus; IABP: intra-aortic balloon pump; OR: odds ratio

Risk Factors	Mehran model OR (95% CI)	p-value	Mehran with NLR OR (95% CI)	p-value
Hypotension	5.42 (1.69 - 17.37)	0.004	5.34 (1.66 - 17.13)	0.005
Use of IABP	4.53 (2.40 - 8.55)	< 0.001	4.43 (2.34 - 8.39)	< 0.001
Age > 75 years	1.82 (1.28 - 2.60)	0.001	1.75 (1.22 - 2.50)	0.002
Contrast	1.11 (0.93 - 1.32)	0.220	1.11 (0.93 - 1.32)	0.247
DM	1.86 (1.35 - 2.55)	< 0.001	1.82 (1.33 - 2.51)	< 0.001
CHF	2.13 (1.30 - 3.48)	0.003	2.05 (1.25 - 3.37)	0.005
CKD	1.79 (1.13 - 2.86)	0.014	1.69 (1.06 - 2.70)	0.028
Anemia	1.89 (1.33 - 2.69)	< 0.001	1.83 (1.28 - 2.60)	0.001
NLR > 2.6			1.81 (1.28 - 2.54)	0.001

## Discussion

In this study, NLR was independently associated with CIN and improved the performance of the Mehran score in predicting CIN in patients with AMI. PLR was not independently associated with CIN in our study.

The etiology of CIN in the setting of AMI is complex. Besides the toxicity of contrast agents, abnormal hemodynamic changes associated with AMI may result in decreased renal blood flow, increased thrombogenic state, activation of inflammatory and neurohormonal cascades, and increased oxidative stress. Studies have demonstrated a strong association between inflammation and renal injury. It has been shown that infiltration of damaged tissue by inflammatory cells leads to worsening of renal injury [11].

The NLR has been a special marker of interest due to its role as a prognostic factor for coronary artery disease (CAD), hypertension, chronic kidney disease (CKD), diabetes, heart failure, cerebrovascular disease, peripheral arterial disease, and malignancy [12]. The plausible pathophysiological mechanism for this relationship is the role of neutrophils in mediating the inflammatory response to acute myocardial injury, causing further tissue damage [13]. The release of reactive oxygen species, myeloperoxidase, and proteolytic enzymes facilitate plaque rupture [14-15]. Lymphocytes play a major role in regulating the immune system [16] and inflammation enhances lymphocytic apoptosis [17].

In several prior studies, a high NLR was associated with increased mortality in patients with STEMI [5, 18]. To date, however, only three studies have explored the association of NLR with CIN in patients undergoing PCI for AMI [19-21]. The prospective study by Kurtul et al. specifically studied the subgroup of AMI patients that presented with NSTEMI [19]. In their study, they demonstrated that a high NLR (> 3.46) had a 73% specificity and a 70% sensitivity for CIN (area under the curve: 0.787), similar to our findings of a ROC of 0.73 when we added NLR to the Mehran score. On the other hand, Kaya et al. studied the role of NLR with CIN in the STEMI population undergoing PCI [20]. They demonstrated that a high NLR (> 6.35) had a sensitivity and specificity of 75% and 65%, respectively, with an AUC of 0.763. Similar findings were noted by Yuan et al. in their study of STEMI patients wherein an NLR > 5.71 had a sensitivity and specificity of 74% and 55%, respectively, with an AUC of 0.708 [21].

Although the association between NLR and CIN has been shown before, our study is novel in its attempt to explore the practical utility of this marker as a predictor of CIN and its interaction with other established risk factors. The Mehran score is one of the well-studied risk score systems to predict CIN and has been validated in patients with AMI [22]. Our study shows that the addition of NLR to Mehran score improved the discriminatory capacity of the score. Thus, NLR could be utilized to predict CIN in AMI patients. Improvement in the prediction model would allow earlier implementation of cardioprotective measures in high-risk patients.

In addition, this is the first study (to the best of our knowledge) to report the utility of NLR as an independent predictor of CIN in a combined group of NSTEMI and STEMI patients in the American population. Based on our results, NLR independently predicted CIN with modest accuracy using a cutoff value of 2.6. NLR with this cutoff has a high negative predictive value that could be clinically useful. Other factors significantly associated with CIN were age, anemia, diabetes mellitus, hypertension, LVEF, CHF admission, tachycardia, use of IABP, and cardiac arrest.

Previous studies have linked a high PLR ratio to increased incidence of CIN in AMI [7]. To date, there are only three studies that have evaluated the role of PLR and its association with CIN in AMI patients who underwent PCI. However, two of these were studied in the STEMI subgroup of patients. Sun et al. noted that a PLR cutoff of > 127.5 was associated with a sensitivity and specificity of 76.8% and 69.2% respectively [23]. On the other hand, Velibey et al. demonstrated that a PLR cutoff of > 177.5 had a sensitivity and specificity of 60% and 72%, respectively; however, when they excluded patients with anemia, PLR was not found to have a significant association with CIN [24]. This could be explained by the fact that iron deficiency anemia is associated with thrombocytosis and thus a higher PLR.

In our study, PLR was not found to have an independent correlation with CIN in AMI patients who underwent PCI. We used a cut-off value of 128 as high PLR as per the study by Cho et al. [10]; however, even when a cutoff value of PLR > 177 (as in the study by Velibey et al.) was used, it remained nonsignificant on the multivariate logistic regression model [24]. We are unable to explain this discrepancy in findings. This potentially could be due to differences in the population (American in our study vs Turkish) or different factors included in the regression model (for example, Velibey et al. did not adjust for CHF on admission or use of IABP) [24]. Further prospective studies are needed to validate the role of PLR in the prediction of CIN in AMI patients.

Limitations

Firstly, the inherent limitation of the study is its retrospective design. Secondly, as in other similar studies, variability in NLR and PLR during the course of hospitalization was not accounted for. Thirdly, we were unable to collect data on potential confounding factors, such as the use of ACE-inhibitors/angiotensin receptor blockers (ARBs), statins, N-acetyl cysteine, and the administration of isotonic fluid solutions. Finally, we did not assess for other causes of acute kidney injury associated with hospitalization, such as the use of diuretics, antibiotics, and other nephrotoxic agents.

## Conclusions

Our study showed a significant correlation between NLR and the incidence of CIN in patients with AMI undergoing cardiac catheterization. NLR is a cheap marker and can be readily calculated at the time of presentation in the emergency department. Our study did not show a significant correlation between PLR and incidence of CIN in AMI patients.
